# The Corneal Ectasia Model of Rabbit: A Validity and Stability Study

**DOI:** 10.3390/bioengineering10040479

**Published:** 2023-04-16

**Authors:** Junchao Wei, Rui He, Xiaogang Wang, Yaowen Song, Jinhan Yao, Xiaona Liu, Xin Yang, Weiyi Chen, Xiaona Li

**Affiliations:** 1 College of Biomedical Engineering, Taiyuan University of Technology, Taiyuan 030024, China; 2 School of Ophthalmology, Shanxi Medical University, Taiyuan 030002, China

**Keywords:** keratoconus, animal model, corneal morphology, biomechanical, uniaxial test

## Abstract

Keratoconus is a bilateral progressive degenerative corneal disease characterized by localized corneal thinning and dilatation. The pathogenesis of keratoconus is not fully elucidated. To gain a better understanding of the pathophysiology of this disease and to explore potential treatments, animal models are essential for basic research. Several attempts have been made to establish animal models of corneal ectasia by using collagenase. However, continuous changes of the cornea have not been well-tracked for the model. In this study, corneal morphology and biomechanical behavior in vivo were determined before and after collagenase Ⅱ treatment at 2, 4, and 8 weeks. The elastic modulus and histology of cornea tissues ex vivo were measured at 8 weeks postoperatively. The results showed that the posterior corneal curvature (Km B) increased and central corneal thickness (CCT) decreased after collagenase treatment. The mechanical properties of ectatic corneas weakened significantly and the collagen fiber interval in the stromal layer was increased and disorganized. This study provides insights into the changes of corneal morphology and biomechanical properties in a rabbit model of corneal ectasia. Changes observed at 8 weeks indicated that the cornea was still undergoing remodeling.

## 1. Introduction

Keratoconus (KC) is a progressive dilated cornea disease characterized by localized corneal thinning, varying degrees of conical deformation, and a high irregularity of myopia and astigmatism [1]. It is a common clinical disorder worldwide with an incidence rate of approximately 1/2000 [2]. KC typically occurs in adolescence and might ultimately resulting in blindness [3]. Clinical treatments for KC include the use of contact lenses, rigid gas permeable lenses, corneal collagen crosslinking, intracorneal ring segment insertion, lenticule implantation, and corneal transplantation [4]. However, the exact etiology of KC remains unknown [5,6,7]. Investigating the underlying mechanism of KC pathogenesis will provide potential treatments for this disease.

Studies have shown that the development of KC is associated with overexpression of degradative enzymes and the reduction of collagen within the stroma [8]. To better understand the occurrence and progression of KC and verify hypotheses regarding its pathogenesis, several teams have generated in vivo animal models of corneal ectasia by infiltrating collagenase or injecting corneal stroma [9,10,11,12,13,14,15]. A significant increase in corneal curvature and a decrease in central corneal thickness (CCT) were observed in these studies after collagenase treatment at a certain time point (e.g., 7 days and 14 days [11,12,13]; 2 months [14]; 3 months [15]). Only Qiao et al. evaluated corneal mechanical behaviors in vivo using Corvis ST. They found deformation amplitude maximum (DA Max) increased significantly at 14 days [11]. The elastic modulus of ectatic cornea decreased at 2 or 3 months [14,15]. The changes in the animal model are consistent with the clinical features of KC and can be utilized for basic investigations of KC. However, the temporal changes in corneal morphology and mechanical properties are still unclear, which are important for understanding the validity and stability of the KC animal model.

In this study, collagenase Ⅱ was applied to establish a rabbit animal model of corneal ectasia. The changes in corneal morphology and biomechanical behaviors in vivo were evaluated preoperatively and at 2, 4, 8 weeks postoperatively. In addition, the corneal elastic modulus ex vivo and histological staining were examined at 8 weeks postoperatively. By investigating the changes in corneal morphology and biomechanical properties of the animal model, this study can provide a better understanding of the development of KC and establish a new foundation for the diagnosis and therapy of KC. 

## 2. Materials and Methods

### 2.1. Animal Model of Corneal Ectasia Establishment 

In this study, to eliminate the refractive changes associated with age development [16], six Japanese white rabbits, 5~6 months old, weighing 3.0~3.5 kg, were used. The animals were obtained from Shanxi Medical University. Preoperative examination showed no ocular disease in either eye. The experimental protocol was approved by the Ethical Committee of Taiyuan University of Technology (TYUT–202106064).

The rabbits were housed in a controlled environment with a 12 h light/dark cycle. Food and water were available ad libitum. Continuous clinical care (24 h per day/7 days per week) was provided throughout the investigation to ensure timely interventions when required. 

The operation was performed on the right eye. Chloramphenicol eye drops (Di Rui Pharmaceutical Co., Ltd., Changchun, China) were administered daily to the cornea for three days prior to the procedure. To prepare the collagenase solution, collagenase type II powder (Worthington Biochemical Corporation, Lakewood, NJ, USA) was dissolved in Hank’s balanced salt solution (Solarbio, Beijing, China) with 15% dextran (Shanghai yuanye Bio-Technology, Shanghai, China) to achieve final concentration of 5 mg/mL. The animals were anesthetized intramuscularly with a 0.2 ml/kg xylazine hydrochloride injection (Shengda Animal Medicine Co., Ltd., Dunhua, China), and corneal topical anesthesia was induced with proparacaine hydrochloride eye drops (Alcon, Fort Worth, TX, USA). After removing the corneal epithelium, a cotton piece with a 4 mm diameter soaked in 100 μL collagenase type II solution was placed on the center of right cornea for 30 min. Then, the cotton piece was removed, and the cornea was rinsed with 0.9% sodium chloride solution.

After the operation, routine postoperative treatment (levofloxacin hydrochloride eye drops (Yangzijiang Pharmaceutical Group Co., Ltd., Taizhou, China) and tobramycin, dexamethasone eye drops (Alcon, Fort Worth, TX, USA), 4 times daily before 21 days post-operatively) was applied to prevent infection. On 7 days postoperatively, slit-lamp microscopy (Jiangsu Kangjie Medical Devices, Taizhou, China) examinations were conducted, which revealed that none of the animals had corneal or conjunctival infections. Additionally, the corneal clouding disappeared, and complete epithelial healing was observed. For further study, all the in vivo examinations were conducted preoperatively and at 2, 4, and 8 weeks postoperatively. For uniaxial testing and histology examination, animals were sacrificed at 8 weeks postoperatively with air embolization at the ear–vein after anesthesia, followed by immediate testing. For in vivo Pentacam (Oculus, Wetzlar, German), Spectral Domain Optical Coherence Tomography (SD–OCT) (Optovue, Fremont, CA, USA), and Corvis ST (Oculus Inc., Wetzlar, German) tests, experienced researchers who were blinded to the study’s design conducted all examinations. 

### 2.2. Measurement of Corneal Morphology In Vivo

The corneal morphology was evaluated using two techniques, Pentacam and SD-OCT. Pentacam provided measurements of mean keratometry from the anterior corneal surface (Km F), mean keratometry from the posterior corneal surface (Km B), max keratometry from the anterior corneal surface (Kmax F), CCT, posterior corneal elevation (PCE), index of surface variance (ISV), index of vertical (IVA), index of height decentration (IHD) and the corneal volumes at 3, 5, 7, and 10 mm (C. Vol D 3 mm, C. Vol D 5 mm, C. Vol D 7 mm, and C. Vol D 10 mm respectively). The overall CCT and corneal epithelial thickness (CET) were measured by SD–OCT.

### 2.3. Measurement of Corneal Biomechanical In Vivo

The changes in the mechanical behaviors of the cornea in vivo were analyzed using Corvis ST. The following parameters were further analyzed: intra-ocular pressure (IOP), CCT, applanation1 velocity (A1V), radius (HCR), peak distance (HC–PD), maximum inverse radius (InvRadMax), applanation1 deflection amplitude (A1 Defl Amp), highest concavity deflection amplitude (HC Defl Amp), applanation2 deflection amplitude (A2 Defl Amp), deflection amplitude maximum (Defl Amp Max), integrated radius (IR), and deformation amplitude at 2 mm around the center (DAR 2 mm).

### 2.4. Uniaxial Testing

**Figure 1:**
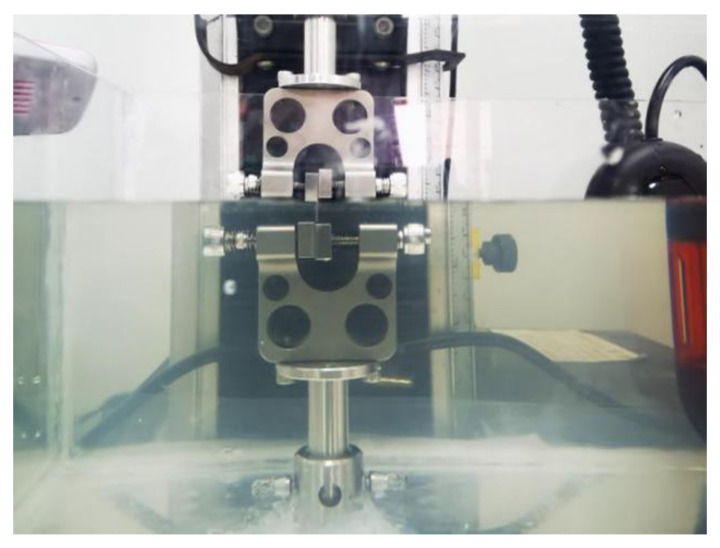
Evaluation of the elastic modulus of corneal strips was carried out by an Instron 5544 extensometer.

To obtain the mechanical properties of the corneal material, the corneal button was cut into a strip with 3 mm width and 12 mm length along the superior–inferior meridian. A commercial stress–strain extensometer (Instron 5544, Instron, Norwood, MA, USA) was used for the uniaxial stretch tests with a 5 N cell. To eliminate pre-stress, samples were preloaded through ten loading and unloading cycles with a magnitude of 0.01 N and a velocity of 2 mm/min before the formal test. During the formal testing, the samples were stretched to 5% strain (in the physiological strain range) at a velocity of 0.5 mm/min. The corresponding stress and strain were measured continuously. The stress was defined as applied force divided by the cross–sectional area (width multiplied by thickness); the strain was defined as an extension over the original length. The thickness of the corneas was obtained by SD–OCT. The elastic modulus, the gradient of the stress–strain curve, was calculated at different stress levels. Samples were immersed in 0.9% saline at room temperature during the whole experiment (Figure 1). To prevent the water surface fluctuation caused by the upward movement of the upper clamp when stretching, which affects the experimental accuracy, the clamp needed to be kept above the liquid level during uniaxial stretching.

### 2.5. Histology

The cornea samples were fixed in 4% paraformaldehyde, then embedded in paraffin and cut into 4 μm–thick sections for hematoxylin–eosin staining and Picrosirius red staining. 

### 2.6. Statistical Analyses

All statistical analyses were performed using SPSS 22.0 (IBM Inc., Chicago, IL, USA). Data that follow a normal distribution are expressed as mean ± standard deviation, while data that do not follow a normal distribution are expressed as the median (lower quartile, upper quartile). A two-tailed paired t-test was used to compare the data in vivo. A two-tailed independent samples’ t-test was used to compare the elastic modulus between the keratoconus and control groups. A *p*–value < 0.05 was considered statistically significant.

## 3. Results

### 3.1. Changes in Corneal Morphology with Time

#### 3.1.1. Morphologic Parameters from Pentacam

**Figure 2:**
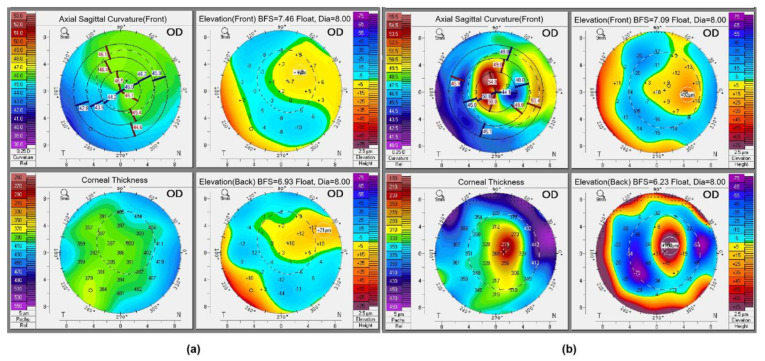
The 4 representative refractive maps of the (**a**) normal cornea and (**b**) ectatic cornea (4 weeks postoperatively) in rabbits obtained from Pentacam.

The four representative refractive maps of the normal cornea and ectatic cornea (4 weeks postoperatively) in rabbits obtained from Pentacam are shown in Figure 2. 

**Figure 3:**
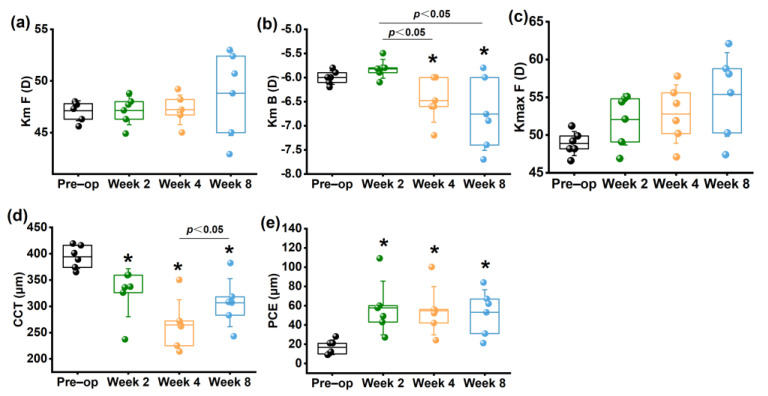
Changes in morphologic parameters measured by Pentacam. (**a**) Km F; (**b**) Km B; (**c**) Kmax F; (**d**) CCT; (**e**) PCE. * *p* < 0.05 vs. preoperative group.

No significant differences were found in Km F at any time point after collagenase treatment (Figure 3a) (*p* > 0.05). Km B showed no significant change at 2 weeks compared to the preoperative group (*p* > 0.05), but increased significantly at 4 and 8 weeks (*p* < 0.05) (Figure 3b). Kmax F increased continuously along the whole follow up period, but showed no significant difference (*p* > 0.05) (Figure 3c).

Compared to the preoperative group, CCT decreased significantly within 8 weeks postoperatively, as shown in Figure 3d. CCT at 4 weeks displayed a significant decrease in comparison to that at 2 weeks (*p* < 0.05). Although there was a slight increase in CCT at 8 weeks (*p* > 0.05), it remained lower than the preoperative group.

The PCE at 2, 4, 8 weeks postoperatively increased significantly compared to the preoperative group (*p* < 0.05). However, there was no significant difference among the postoperative groups (*p* > 0.05), which suggested no sustained change in PCE within 8 weeks after collagenase treatment (Figure 3e).

**Figure 4:**
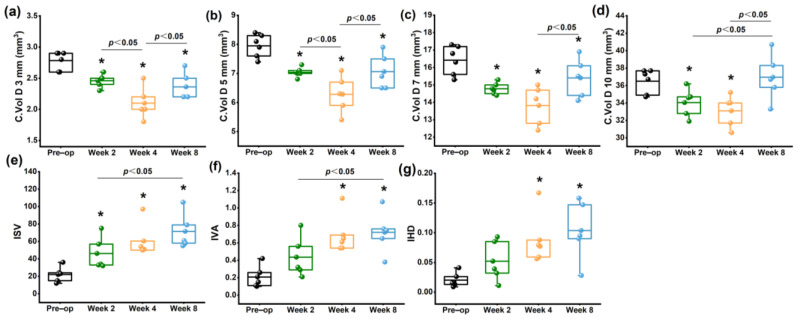
Changes in corneal volume and corneal morphological variation index measured by Pentacam. (**a**) C. Vol D 3 mm; (**b**) C. Vol D 5 mm; (**c**) C. Vol D 7 mm; (**d**) C. Vol D 10 mm; (**e**) ISV; (**f**) IVA; (**g**) IHD. * *p* < 0.05 vs. preoperative group.

The C. Vol D 3 mm (Figure 4a), C. Vol D 5 mm (Figure 4b), C. Vol D 7 mm (Figure 4c) and C. Vol D 10 mm (Figure 4d) diameter discs were significantly decreased within 8 weeks after collagenase treatment, particularly in the central and paracentral area (*p* < 0.05). At 8 weeks, the corneal volume was increased significantly compared to the 4 week groups (*p* < 0.05). The corneal morphological variation index, including ISV (Figure 4e), and IVA (Figure 4f), and IHD (Figure 4g), gradually increased with time after collagenase treatment, with significant differences observed at 4 and 8 weeks (*p* < 0.05). Detailed informations were shown in Supplementary Table S1.

#### 3.1.2. Morphologic Parameters from SD–OCT

**Figure 5:**
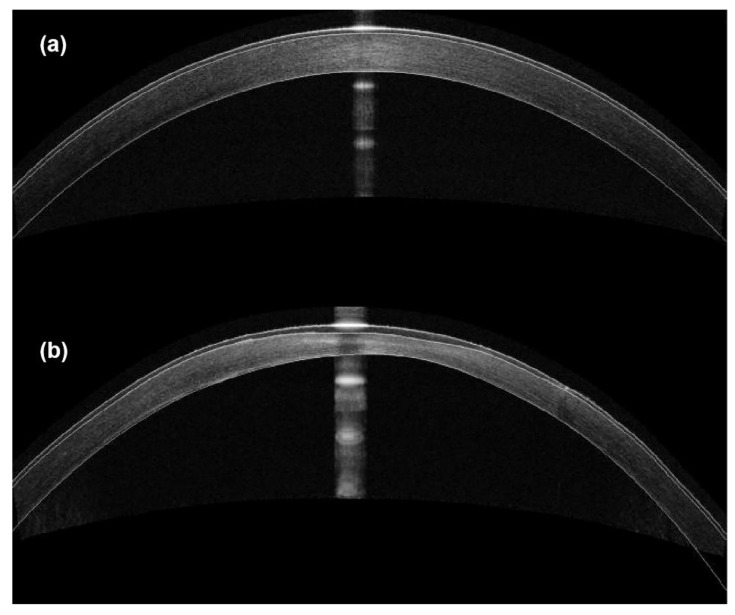
The representative SD-OCT images of the cornea (**a**) preoperatively and (**b**) at 4 weeks postoperatively.

**Figure 6:**
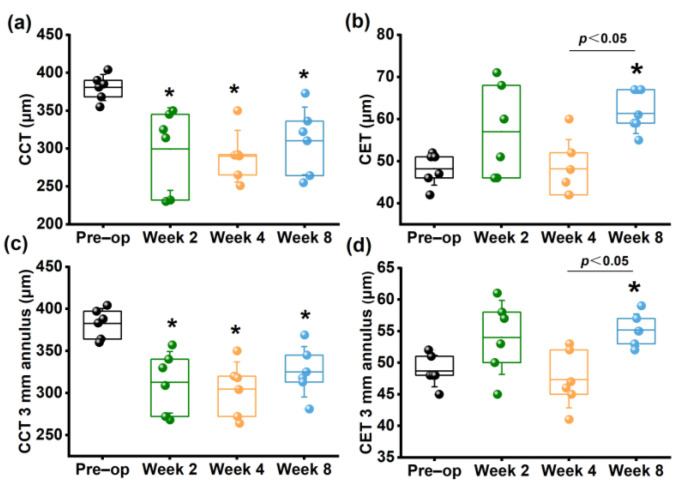
Changes in morphologic parameters measured by SD-OCT. (**a**) CCT; (**b**) CET; (**c**) CCT 3 mm annulus; (**d**) CET 3 mm annulus. * *p* < 0.05 vs. preoperative group.

The representative SD-OCT images of rabbit corneas are shown in Figure 5. Obvious increases in corneal curvature and a decrease in corneal thickness within the 3 mm diameter were observed. As shown in Figure 6, CCT (Figure 6a) and average thickness within the 3 mm diameter annulus (Figure 6c) continuously decreased within 4 weeks postoperatively compared to the preoperative group (*p* < 0.05). Although they increased at 8 weeks compared to 2 and 4 weeks postoperatively, they were still significantly lower than those of the preoperative group (*p* < 0.05). These results were consistent with the CCT measured by Pentacam.

Thickening of the corneal epithelium at the corneal apex (CET) (Figure 6b) and within the 3 mm diameter annulus (Figure 6d) were observed at 2 weeks and disappeared at 4 weeks, but no significant difference was found compared to the preoperative group (*p >* 0.05). However, at 8 weeks, there was a significant increase compared to the preoperative CET (*p* < 0.05). Detailed information were shown in Supplementary Table S2.

### 3.2. Changes in Corneal Mechanical Properties with Time

#### 3.2.1. Corneal Mechanical Behaviors Determined by Corvis ST In Vivo

**Figure 7:**
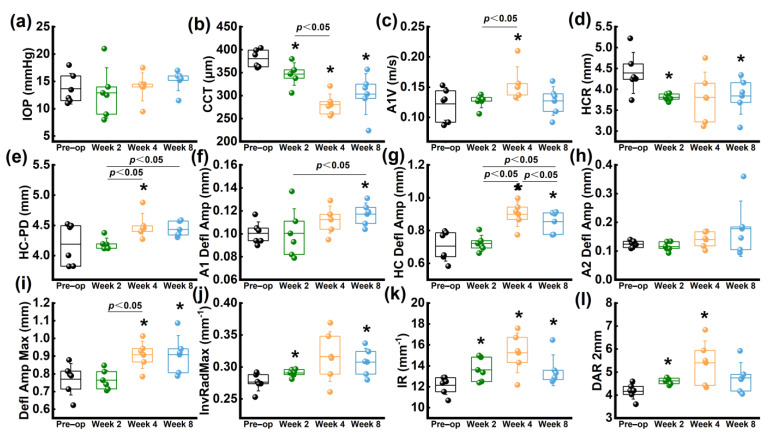
The parameters derived by Corvis ST. (**a**) IOP; (**b**) CCT; (**c**) A1V; (**d**) HCR; (**e**) HC−PD; (**f**) A1 Defl Amp; (**g**) HC Defl Amp; (**h**) A2 Defl Amp; (**i**) Defl Amp Max; (**j**) InvRadMax; (**k**) IR; (**l**) DAR 2 mm. * *p* < 0.05 vs. preoperative group.

There was no significant change observed in IOP during the entire follow-up period (*p* > 0.05), suggesting that the collagenase treatment did not result in any abnormality in IOP levels (Figure 7a). Moreover, the trend of CCT changes measured by Corvis ST was consistent with the findings from Pentacam and SD–OCT (Figure 7b).

Within 4 weeks after the operation, there was a significant increase in A1V compared to the preoperative group, followed by a decrease at 8 weeks (Figure 7c). The HCR showed a decreasing trend throughout the follow-up period (Figure 7d); while HC–PD increased within 4 weeks postoperatively and decreased at 8 weeks (Figure 7e). Both A1 Defl Amp (Figure 7f), HC Defl Amp (Figure 7g) and Defl Amp. Max (Figure 7i) increased within 8 weeks, with only a statistical difference observed at 8 weeks (*p* < 0.05). 

Compared to the preoperative group, InvRadMax (Figure 7j) and IR (Figure 7k) increased significantly within 4 weeks postoperatively (*p* < 0.05), while a decreasing trend was observed at 8 weeks. Similarly, DAR 2 mm (Figure 7l) also increased significantly at 2 and 4 weeks (*p* < 0.05), but a decreasing trend was observed at 8 weeks. Detailed informations were shown in Supplementary Table S3.

#### 3.2.2. Corneal Elastic Modulus Determined by Uniaxial Tension Ex Vivo

**Figure 8:**
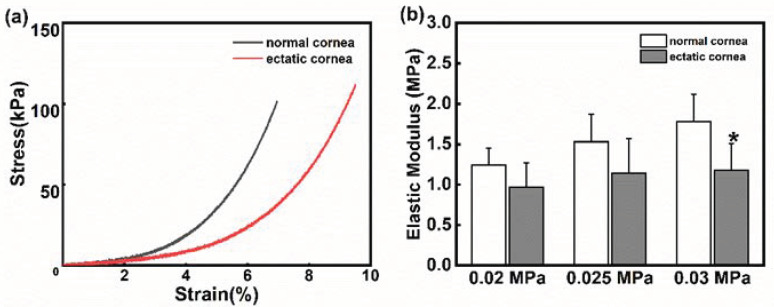
(**a**) The stress–strain curves of the normal cornea (gray line) and the ectatic cornea (red line); (**b**) Changes in the elastic modulus in the normal cornea and ectatic cornea at different stress levels. * *p* < 0.05 vs. normal cornea.

The stress–strain curves of the normal cornea and the ectatic cornea at 8 weeks are shown in Figure 8a. It could be observed that the stress in the normal group was higher than that of the ectatic cornea at the same strain level, suggesting that the mechanical properties of the ectatic cornea were weakened.

Similar to other biomaterials, the cornea shows a varying elastic modulus under different physiological conditions. As Figure 8b shows, the elastic modulus improved as the stress increased, and the differences between the normal cornea and ectatic cornea were not statistically significant at 0.02 MPa and 0.025 MPa stresses (*p* < 0.05). However, the elastic modulus of the ectatic cornea was significantly lower than that of the normal cornea at 0.03 MPa stress (*p* < 0.05).

### 3.3. H&E Staining and Picrosirius-Red Staining

**Figure 9:**
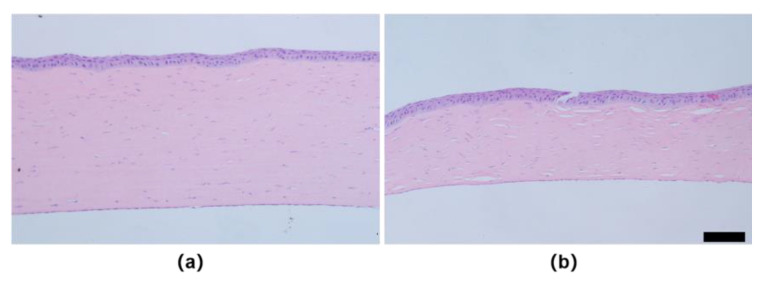
The H&E staining corneal sections of rabbits at 8 weeks. (**a**) normal cornea; (**b**) ectatic cornea (The scale bar is 100 μm).

**Figure 10:**
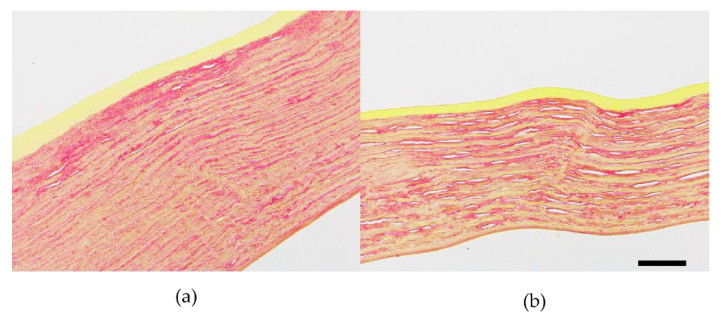
The Picrosirius red staining corneal sections of rabbits at 8 weeks. (**a**) normal cornea; (**b**) ectatic cornea (The scale bar is 100 μm).

Histological staining revealed that both normal and ectatic corneal tissues appeared intact, with well-repaired epithelial layers and basically complete endothelial layers at 8 weeks. However, the ectatic cornea showed a significant reduction in corneal thickness and an increase in disorganized collagen fiber intervals in the stromal layer (Figure 9). Additionally, as shown in Figure 9b, there was a decrease in the total number of cells in the stromal layer of the ectatic cornea. The Picrosirius red staining of ectatic cornea (Figure 10b) showed less collagen content and fewer tightly packed collagen fibers compared with the normal cornea (Figure 10a).

## 4. Discussion 

Several studies have established animal models of corneal ectasia that exhibit clinical KC features, such as decreased CCT and increased corneal curvature [9,10,11,12,13,14,15]. However, the changes in morphology and biomechanics in ectatic corneas over time are still unknown. In the present study, we found that CCT and biomechanical properties continuously decreased at 2 and 4 weeks, then showed an increasing trend at 8 weeks. However, they were still significantly lower than before treatment, suggesting that the morphological and mechanical properties of the cornea tissue were still being remodeled within 8 weeks. This model effectively mimicked changes in the morphology and mechanical properties of KC, but it was still not stable at 8 weeks postoperatively.

KC is a localized corneal ectasia disease, typically with a protrusion occurring within a region of 2~5 mm in diameter, in the central or paracentral cornea [17]. In previous studies, collagenase was applied on a large area of the cornea to induce almost full-corneal ectasia. Only Hu et al. caused a 6 × 6 mm^2^ collagenase region through the intra-stromal injection of type I collagenase in New Zealand white rabbits [14]. In this study, collagenase was applied to the central 4 mm diameter surface of the cornea, which was similar in size to the cone-shaped corneal region in KC patients. Intra-stromal injection is commonly used to treat infectious corneal disease. Drugs can be delivered to the stroma from the corneal limbus into multifocal infection, without disrupting the corneal epithelium. However, this method may cause mechanical damage to corneal tissue during the needle piercing. In addition, the injection depth is not easy to determine, and drug leakage may also be inevitable. Although collagenase infiltration requires removal of the corneal epithelium, it is a simple and convenient way to achieve topical corneal dilation, and the drug can be locally held. 

It is well-known that changes in corneal thickness play a crucial role in the grading criteria of KC [18]. Previous studies have reported a decrease in CCT within three months following collagenase treatment [9,10,11,12,13,14,15]. In this study, continuous decreases were observed in terms of the CCT measured by Pentacam, SD–OCT, or Corvis ST for 4 weeks, while it began to increase at 8 weeks. These findings indicate that the corneal tissue started self-repairing between 4 to 8 weeks after collagenase treatment. 

SD–OCT is a non–invasive imaging technique used to measure corneal thickness, including the thickness of the corneal epithelium. Numerous studies revealed corneal epithelial thinning around the dilated region in KC [19,20,21], whereas others reported no significant change [22] or even an increase in epithelial thickness in KC [23,24]. Additionally, Sandali et al. demonstrated that stromal loss often occurred in the location of the cone in the advanced stage of KC, with stromal thinning [25]. Therefore, the epithelium becomes thickest in this region to compensate for the corneal thickness. However, these studies have not measured the corneal epithelial thickness after establishing the animal model of corneal ectasia. In this study, SD–OCT data showed that the corneal epithelium was significantly thickened at 2 weeks postoperatively, possibly related to the self–repair of corneal tissue after epithelial scraping. CET then returned to the preoperative level at 4 weeks and it began to increase at 8 weeks postoperatively to provide a smooth optical surface. The change of CET throughout the follow–up period was approximately 10 µm, which was about one-fifth of the change in the total corneal thickness. This suggests that changes in the stromal layer of the cornea contribute a major part of the variation in corneal thickness.

The variation in corneal curvature is associated with the refractive power of the eye and relates to many refractive disorders. The clinical investigation demonstrated that anterior or posterior curvatures varied in patients with KC [26]. PCE and Km B are important indicators of KC in clinical practice [27,28,29]. Previous studies have shown that the application of collagenase can lead to an increase in anterior corneal curvature [10,11,12,13,14,15]. In our study, after collagenase treatment, there was no significant change in Km F, while Km B and PCE increased, suggesting that the posterior corneal surface occurred a forward protrusion. In addition, Ahmadi et al. have found that C. Vol D 3 mm, C. Vol D 5 mm, C. Vol D 7 mm, and C. Vol D 10 mm in KC patients were significantly smaller than normal controls, particularly in the central and paracentral areas. The degree of the reduction was thought to be with the severity level of the disease [30,31]. The decrease in corneal volume could be explained by the loss of corneal tissue. In this study, we found that C. Vol D 3 mm, C. Vol D 5 mm, C. Vol D 7 mm, and C. Vol D 10 mm continued to decrease within 4 weeks and increased at 8 weeks. This suggested that the corneal loss occurred within 4 weeks and the corneal volume increased at 8 weeks. Furthermore, we observed that ISV, IVA, and IHD also changed continuously up to 8 weeks. These results indicated that the turning point of morphological change might occur between 4 and 8 weeks after collagenase treatment. 

Biomechanical properties play a critical role in corneal tissue, especially in the case of ectatic diseases [32]. Corvis ST is based on the Scheimpflug high-speed imaging technology and is capable of capturing 140 images in 31 ms. It can provide dynamic corneal response (DCR) parameters when an air puff is applied to the cornea. Corvis ST has good repeatability and reliability in determining biomechanical behaviors [33]. It has been often applied to distinguish KC from normal eyes, combined with morphological detection [34,35,36,37]. Several parameters, such as A1V, DAR 2 mm, IR, stiffness parameter at first applanation (SP-A1), and Ambrosio’s relational thickness horizontal (ARTh), which could derive a combined parameter Corvis biomechanical index (CBI), have been found to be significant in determining KC [34]. In addition, other parameters such as HCDA, HCR, and A1T also be considered effective in determining KC in clinical practice [35,36,37]. However, some studies pointed out that some parameters (e.g., A2T, HCT) derived by Corvis ST for KC detection are of poor reproducibility and instability of measurement and could not be used as sensitive discriminators to distinguish the normal cornea and KC, especially for forme fruste keratoconus (FFKC) [36]. The differences in the in vivo biomechanical properties of patients with KC may be related to sample size, age, disease severity, and test reproducibility. Previous studies reported that IR and DAR 2 mm increase with the softening of the cornea [33,38]. In this study, a significant increase in IR at any time point after collagenase application indicated that the corneas became softer and less resistant to deformation, while at 8 weeks, IR began to decrease, which indicated that the cornea regained its resilience. Similarly, DAR 2 mm increased gradually at 2 and 4 weeks, indicating that the cornea gradually became softer and the greater deformation occurred mainly in the central cornea, and less in the paracentral cornea; then, it raised at 8 weeks. Changes of IR and DAR 2 mm indicated that the mechanical properties of the cornea became weakened at 2 and 4 weeks, and rebounded at 8 weeks. The variations of these parameters suggested that the cornea’s capacity to resist the deformation reduced after collagenase treatment. 

The normal cornea undergoes only small deformation when subjected to an air puff. Due to the abnormal structure and weakened mechanical properties in KC, the cornea is more susceptible to deformation under external forces. Interestingly, changes in several parameters, such as A1V, InvRadMax, IR, and DAR 2 mm, indicated that the corneal mechanical properties in vivo were enhanced at 8 weeks postoperatively. Combined with the Pentacam and SD-OCT results, this phenomenon might be related to the thickening of the cornea, as well as indicating tissue repair of the cornea. Moreover, corneal scarring was often observed in advanced stages of KC in the clinic [25]. In this study, increasing the optical density was observed in ectatic corneas in the SD–OCT images. A variable amount of stromal opacities observed in ectatic corneas at 8 weeks might result in an increase in CCT and improved biomechanical properties.

Collagen degradation and tissue structure destruction in KC contribute to the reduction of corneal material mechanical properties [39,40,41]. In previous ectatic corneal animal models, compared to the normal cornea, the elastic modulus of the ectatic cornea reduced significantly at 15~20% strain level [12,14,15], while no significant difference was found under a 5% strain level at 3 months after the collagenase treatment. In this study, we measured the corneal elastic modulus at three stress levels within the physiological condition, which was more representative of the real physical environment of the cornea. The elastic modulus of ectatic corneas decreased at 0.02 and 0.025 MPa, with no statistical significance, while it was significantly lower than that of normal corneas at the 0.03 MPa stress level. Less content and disorganized collagen fibers in ectatic corneas by histological staining also supported the uniaxial stretch results. Overall, changes in the mechanical properties of the ectatic cornea in vivo and ex vivo both demonstrated the insufficient ability to resist deformation in the ectatic cornea. For this reason, the cornea of KC patients is more prone to protrusion. 

The development of an ectatic corneal animal model has been instrumental in advancing our understanding of the underlying mechanisms of KC and in exploring potential treatments, such as corneal collagen crosslinking (CXL), lamellar keratoplasty, and potential therapeutic drug. Besides investigating morphological and mechanical changes in the cornea [9,10,11,12,13,14,15], Qiao et al. also found that the markers of oxidative stress increased after collagenase treatment, and sulforaphane could protect rabbit KC against oxidative stress injury [12]. Hu et al. performed CXL on ectatic corneas caused by collagenase and found an increase in the corneal elastic modulus after crosslinking [14]. Clinical studies have found elevated expressions of inflammatory mediators in the corneas of KC patients [42]. Increasing levels of inflammatory molecules could induce the expression of protease and degradation of collagen, which is associated with the severity of KC. The application of drugs such as cyclosporine A (CyA) to reduce MMP–9 levels in tears has shown promise in slowing down or preventing the progress of KC [43]. In future studies, cytokines, including the inflammatory mediators in animal ectatic cornea, will be explored to find whether this model is consistent with the pathology of KC in clinic, and to provide a potential target for KC treatment.

This study has limitations that should be acknowledged. Firstly, the use of collagenase in this study to induce cornea ectasia is an acute chemical injury and may not fully mimic the pathogenesis of KC. Alternative methods that are more suitable and more effective should be explored to establish this animal model. Secondly, the sample size in this study is limited; the number of animals should be enlarged to improve the reliability of the experiment. Thirdly, the follow–up period used for tracking the changes in corneal morphology and in vivo biomechanical properties was limited to 8 weeks postoperatively. Longer observation periods are needed to fully understand the temporal changes in the ectatic cornea and to verify the study results.

The animal models of mice [9] and rabbits [10,11,12,13,14,15] had been established to mimic the pathological process of KC and could be used for KC research. However, the continuous changes in corneal morphology and biomechanics in vivo have not been thoroughly tracked in these models. In summary, we investigated the validity and stability of the rabbit corneal enzymatic degradation model by conducting follow-up measurements within 8 weeks. Our results confirmed that the ectatic cornea exhibited thinner, more protruding and softer properties, which were consistent with the clinical characteristics of KC. This model effectively mimicked changes in the morphology and mechanical properties of KC, but it was still not stable at 8 weeks postoperatively. Therefore, the ectatic corneal animal model could be used for KC research. In addition, the changes in corneal morphology and in vivo biomechanics at 8 weeks indicated that the ectatic cornea began to self-repair, which requires further investigation.

## Data Availability

All data generated or analyzed during this study are included in this published article.

## Supplementary Materials

The following supporting information can be downloaded at: https://www.mdpi.com/article/10.3390/bioengineering10040479/s1, Table S1: Changes of Pentacam parameters versus to pre-operation group; Table S2: Changes of SD-OCT parameters versus to pre-operation group; Table S3: Changes of Corvis ST parameters versus to pre-operation group.
