# Supplementary material for: The Corneal Ectasia Model of Rabbit: A Validity and Stability Study
Source: Bioengineering (Basel). 2023 Apr 16;10(4):479. doi: 10.3390/bioengineering10040479 (PMC10135747; doi:10.3390/bioengineering10040479)
Supplement: Supplementary file 1 [file bioengineering-10-00479-s001.zip › bioengineering-2282844-supplementary.pdf]

bioengineering MDPI

Supplementary Materials

Table S1. Changes of Pentacam parameters versus to pre–operation group.

	Pre–op	Week 2		Week 4		Week 8	
	Mean±SD	Mean±SD	P–value	Mean±SD	P–value	Mean±SD	P–value
**Km F,D**	47.1±0.9	47.1±1.4	0.975	47.2±1.4	0.878	48.8±4.1	0.369
**Km B,D**	-6±0.1	-5.8±0.2	0.187	-6.5±0.4	0.047	-6.7±0.7	0.045
**Kmax F,D**	48.9±1.59	52.1±3.39	0.099	52.8±3.87	0.079	55.4±5.54	0.054
**CCT,µm**	373±18.9	244.4±56.6	0.002	244±45.8	0	289±40.0	0.003
**PCE,µm**	16.8±7.6	57.6±27.8	0.03	54.8±25.1	0.014	53±23.4	0.016
**C.Vol D 3mm, mm^3**	2.85（2.6, 2.9）	2.46±0.10	0.028	2.1±0.24	0.027	2.36±0.21	0.026
**C.Vol D 5mm, mm^3**	7.95±0.39	7.04±0.16	0.003	6.28±0.59	0	7.06±0.56	0.001
**C.Vol D 7mm, mm^3**	16.42±0.8	14.78±0.33	0.001	13.82±1.03	0.001	15.4±1.04	0.015
**C.Vol D 10mm, mm^3**	36.5±1.37	34.04±1.5	0	33.1±1.69	0.003	36.96±2.47	0.551
**ISV**	22±8.36	46.2±17.15	0.04	52.5（50, 69.55）	0.028	71.6±18.67	0.002
**IVA**	0.21±0.12	0.43±0.22	0.099	0.63（0.54, 0.79）	0.028	0.72±0.22	0.002
**IHD**	0.02±0.01	0.05±0.03	0.098	0.07（0.05, 0.10）	0.028	0.10±0.05	0.007

Table S2. Changes of SD–OCT parameters versus to pre–operation group.

	Pre–op	Week 2		Week 4		Week 8	
	Mean±SD	Mean±SD	P–value	Mean±SD	P–value	Mean±SD	P–value
**CCT, µm**	380.5±17.1	299.3±54.5	0.01	289.8±33.9	0	310±44.5	0.004
**CET, µm**	48.1±3.8	57±11	0.073	48.1±6.9	1	61.3±4.8	0.001
**CCT 3mm annulus, µm**	382.7±16.7	312.7±36.5	0.003	304.7±32.2	0.001	325.2±30	0.003
**CET 3mm annulus, µm**	48.7±2.5	54±5.9	0.135	47.3±4.5	0.379	55.2±2.6	0.015

Table S3. Changes of Corvis ST parameters versus to pre–operation group.

	Pre–op	Week 2		Week 4		Week 8	
	Mean±SD	Mean±SD	P–value	Mean±SD	P–value	Mean±SD	P–value
**IOP, mmHg**	13.67±2.78	12.9±4.61	0.748	14±2.57	0.852	15.2±1.91	0.39
**CCT, µm**	380.3±19.3	347±24.48	0.022	280.2±23.3	0	303.4±44.54	0.004
**A1V, m/s**	0.12±0.02	0.13±0.01	0.756	0.15 (0.13,0.17)	0.046	0.13±0.02	0.803
**HCR, mm**	4.39±0.49	3.81±0.08	0.048	3.81±0.60	0.141	3.84±0.44	0.039
**HC–PD, mm**	4.19±0.34	4.19±0.1	0.981	4.49±0.20	0.042	4.43±0.12	0.096
**A1 Defl Amp, mm**	0.10±0.01	0.10±0.02	0.989	0.11±0.01	0.093	0.12±0.01	0.005
**HC Defl Amp, mm**	0.70±0.09	0.72±0.05	0.691	0.90±0.07	0.001	0.85±0.06	0.014
**A2 Defl Amp, mm**	0.12±0.01	0.12±0.01	0.14	0.14±0.03	0.307	0.18±0.1	0.211
**Defl Amp Max, mm**	0.77±0.09	0.76±0.05	0.894	0.91±0.07	0.004	0.91±0.11	0.009
**InvRadMax, mm^{-1}**	0.27±0.014	0.29±0.01	0.03	0.32±0.04	0.076	0.31±0.02	0.003

**IR, mm^{-1}**	12.14±0.87	13.61±1.11	0.013	15.24±1.89	0.007	13.1（12.64,14.3）	0.046
**DAR 2 mm**	4.17±0.34	4.62±0.14	0.008	5.41±0.94	0.028	4.75±0.66	0.153
